# Killed Whole-Cell Oral Cholera Vaccine Induces CCL20 Secretion by Human Intestinal Epithelial Cells in the Presence of the Short-Chain Fatty Acid, Butyrate

**DOI:** 10.3389/fimmu.2018.00055

**Published:** 2018-01-29

**Authors:** Ju-Ri Sim, Seok-Seong Kang, Daesang Lee, Cheol-Heui Yun, Seung Hyun Han

**Affiliations:** ^1^Department of Oral Microbiology and Immunology, DRI, and BK21 Plus Program, School of Dentistry, Seoul National University, Seoul, South Korea; ^2^Department of Food Science and Biotechnology, Dongguk University Seoul, Goyang, South Korea; ^3^The 5th R&D Institute, Agency for Defense Development, Daejeon, South Korea; ^4^Department of Agricultural Biotechnology and Research Institute for Agriculture and Life Sciences, Seoul National University, Seoul, South Korea

**Keywords:** cholera vaccine, short-chain fatty acids, butyrate, chemokines, intestinal epithelial cells

## Abstract

Short-chain fatty acids (SCFAs), such as acetate, butyrate, and propionate, modulate immune responses in the gut. However, the effect of SCFAs on mucosal vaccine-induced immune cell migration is poorly understood. Here, we investigated whether SCFAs modulate chemokine expression induced by the killed whole-cell oral cholera vaccine, Shanchol™, in human intestinal epithelial cells. Shanchol™ induced expression of CCL2, CCL5, CCL20, and CXCL10 at the mRNA level, but not at the protein level. Interestingly, CCL20 secretion was substantially increased by co-stimulation with Shanchol™ and butyrate, while neither acetate nor propionate showed such effect. Enhanced CCL20 secretion was associated with GPR109A activation, and histone deacetylase (HDAC) inhibition. In addition, co-treatment with Shanchol™ and butyrate synergistically increased the secretion of adenosine triphosphate (ATP). Moreover, CCL20 secretion was decreased by inhibiting the extracellular ATP receptor P2X7. However, neither inflammasomes nor caspases were involved in CCL20 production. The culture supernatant of cells treated with Shanchol™ and butyrate augmented human immature dendritic cell migration. Collectively, these results suggest that butyrate enhances Shanchol™-induced CCL20 production in human intestinal epithelial cells *via* HDAC inhibition and ATP-P2X7 signaling by activating GPR109A. These effects potentially enhance the mucosal immune responses in the gut induced by this oral cholera vaccine.

## Introduction

Cholera is an acute diarrheal disease that can cause life-threatening dehydration and shock if not treated appropriately. It is caused by ingestion of water or food contaminated with *Vibrio cholerae* (1). Currently, only killed whole-cell-based oral cholera vaccines are commercially available. Dukoral™ is formulated with heat-killed or formalin-killed *V. cholerae* O1 together with the recombinant cholera toxin B subunit (CTB) (2). Shanchol™ includes formalin-killed *V. cholerae* O139 and heat-killed or formalin-killed *V. cholerae* O1, but does not include the recombinant CTB subunit (3). Although both cholera vaccines have been successfully licensed, their low immunogenicity, short-term protection, and high dose requirement leave a room for further improvement (4, 5). Enhancement of intestinal mucosal immunity has been suggested to be one of the most efficient approaches by which the development of modern oral vaccines against cholera can be improved (6).

Mucosal immune responses induced by oral cholera vaccines are mainly mediated by anti-bacterial and anti-cholera toxin antibodies in the mucosal compartments of gastrointestinal tract (7). The gastrointestinal tract has gut-associated lymphoid tissues (GALTs), such as Peyer’s patches, which consist of antigen-presenting cells (APCs) and lymphocytes, both of which play a crucial role in the mucosal immune system (8). Following oral vaccination, antigens in the mucosal inductive site can be sampled by M cells and transported to APCs or directly captured by APCs such as dendritic cells (DCs). In Peyer’s patches, antigen-loaded DCs migrate to T-cell areas and subsequently present the antigen to T cells in GALTs and the mesenteric lymph nodes. Finally, IgA-producing plasmablasts home to the effector site, after which antigen-specific dimeric IgA antibodies are produced and transported to the lumen.

Epithelial cells in the gastrointestinal tract induce mucosal immune responses by producing immune mediators such as chemokines (9). Chemokines play a central role in the mucosal immunity by regulating the patterns of leukocyte chemotactic migration. For example, CCL25 is highly expressed in the small intestine where it supports lymphocyte homing (10). Besides, in the large intestine, CCL28, also called mucosa-associated epithelial chemokine, is expressed and plays a key role in the recruitment of IgA antibody-secreting cells (11). CCL20, also known as macrophage inflammatory protein-3α, binds to CC chemokine receptor-6 (CCR6) (12) and attracts immature DCs (13), memory T cells (14), and B cells (15). In addition, increased number of DCs induced by CCL20 upon mucosal vaccination is associated with the extent to which IgA and IgG levels in the nasal mucosa are elevated (16), indicating that chemokine induction is important for efforts to improve vaccine efficacy.

Short-chain fatty acids (SCFAs), such as acetate, butyrate, and propionate, are the major metabolites of dietary fibers derived from the intestinal microbiota. These metabolites can regulate intestinal immune responses by enteric pathogens (17). SCFAs modulate immune cell function by inhibiting the activity of histone deacetylase (HDAC), which regulates epigenetic modification, or by activating G-protein-coupled receptors (GPCRs) (18–20), thus modulating chemokine production and release (21). Although SCFAs are the major end products of gut microbiota in the large intestine, they are also found in the small intestine (22). In addition, SCFAs increase the number of IgA^+^ plasma cells in the small intestine (23). Since microbial metabolites, including SCFAs in the gut, can affect mucosal vaccine efficacy (24), they would be employed as vaccine adjuvants possibly by being delivered together with vaccines, or by being elevated in the gut with high fiber diet prior to vaccination. However, the effects of SCFAs on mucosal vaccine-induced immune responses, particularly chemokine expression, are not clearly understood. In this study, we investigated whether SCFAs modulate Shanchol™-induced chemokine expression in human intestinal epithelial cells, with the aim of further improving vaccine efficacy.

## Materials and Methods

### Reagents and Chemicals

The killed whole-cell oral cholera vaccine, Shanchol™, was purchased from Shantha Biotechnics (Hyderabad, India). Sodium acetate, sodium butyrate, sodium propionate, adenosine triphosphate (ATP), trichostatin A (TSA), mepenzolate bromide (MPN), and oxATP were obtained from Sigma-Aldrich Inc. (St. Louis, MO, USA). SB203580, PD98059, and SP600125 were purchased from Calbiochem (La Jolla, CA, USA). For Western blot analysis, a monoclonal antibody specific to acetyl-histone H3 (Lys9) was obtained from Cell Signaling Technology (Beverly, MA, USA). For chromatin immunoprecipitation (ChIP) assay, a polyclonal antibody against acetyl-histone H3 was purchased from Millipore (Billerica, MA, USA). Anti-CCL20-neutralizing antibodies and mouse IgG isotype control were purchased from R&D Systems (Minneapolis, MN, USA). Phycoerythrin-conjugated anti-human CD196 (CCR6) antibody and its isotype control were purchased from Biolegend (San Diego, CA, USA).

### Cell Cultures

The human intestinal epithelial cell lines Caco-2 and HT-29 were cultured in Dulbecco’s modified Eagle medium (Hyclone, Logan, UT, USA) supplemented with 10% heat-inactivated fetal bovine serum (FBS) (Gibco, Burlington, ON, Canada), 100 U/ml penicillin, and 100 µg/ml streptomycin (Hyclone). Cells were grown at 37°C in a 5% CO_2_-humidified incubator. Polarized Caco-2 cells were prepared as described previously (25). The human intestinal epithelial cell line SNU-407 was purchased from the Korean Cell Line Bank (Seoul, Korea) and maintained in Roswell Park Memorial Institute 1640 medium (Hyclone) containing 10% FBS, 100 U/ml penicillin, and 100 µg/ml streptomycin at 37°C in a 5% CO_2_-humidified incubator.

### Reverse Transcription-Polymerase Chain Reaction (RT-PCR)

Total RNA was isolated using TRIzol reagent (Invitrogen, Carlsbad, CA, USA) according to the manufacturer’s instruction. Complementary DNA (cDNA) was synthesized using random hexamers (Roche, Basel, Switzerland) and M-MLV reverse transcriptase (Promega, Madison, WI, USA). Amplification of cDNA was performed by PCR in a total volume of 20 µl containing EmeraldAmp PCR Master Mix (Takara Biomedical Inc., Osaka, Japan) and 10 pmol of primers specific for human chemokines or β-actin. The PCR conditions to amplify all chemokine genes used in this study were initial denaturation at 95°C for 5 min; amplification through 32 or 35 cycles of 95°C for 40 s, 60°C for 40 s, and 72°C for 40 s; and final extension at 72°C for 7 min. The PCR conditions for β-actin amplification were initial denaturation at 95°C for 5 min; amplification by 24 cycles of 95°C for 30 s, 55°C for 30 s, and 72°C for 3 min; and a final extension at 72°C for 10 min. Real-time RT-PCR was performed with an Applied Biosystems 7500 real-time PCR system (Applied Biosystems, Waltham, MA, USA) as described previously (26). The sequences of the human chemokine-specific primers were as follows: CCL2, forward 5′-TCC CCA GAC ACC CTG TTT TA-3′ and reverse 5′-CAA AAC ATC CCA GGG GTA GA-3′; CCL5, forward 5′-GAA AGA ACC GCC AAG TGT GT-3′ and reverse 5′-GTA GAA TCT GGG CCC TTC AA-3′; CCL20, forward 5′-GCC AAT GAA GGC TGT GAC AT-3′ and reverse 5′-AAC CCC AGC AAG GTT CTT TC-3′; CXCL10, forward 5′-GAT GTT CTG ACC CTG CTT CA-3′ and reverse 5′-GAA AGA ATT TGG GCC CCT TG-3′ and; CCL25, forward 5′- GTC CAC ACC CAA GGT GTC TT-3′ and reverse 5′-TGT AGG GCG ACG GTT TTA TC-3′ and; CCL28, forward 5′-GCT GAT GGG GAT TGT GAC TT-3′ and reverse 5′-GTT TCG TGT TTC CCC TGA TG-3′ and; β-actin, forward 5′-GTG GGG CGC CCC AGG CAC CA-3′ and reverse 5′-CTC CTT AAT GTC ACG CAC GAT TTC-3′; GAPDH, forward 5′-AAG GTG AAG GTC GGA GTC AA-3′ and reverse 5′-ATG ACA AGC TTC CCG TTC TC-3′.

### Determination of Chemokine Production Using an Enzyme-Linked Immunosorbent Assay (ELISA)

Caco-2 cells or other human intestinal epithelial cells were stimulated with the indicated stimuli, the cell culture supernatants were collected, and their chemokine contents were determined using the appropriate ELISA kit (R&D or Biolegend) according to the manufacturer’s instructions.

### Measurement of ATP Secretion

Caco-2 cells (4 × 10^5^ cells/ml) were treated with Shanchol™ and/or butyrate for 2 h. The concentrations of extracellular ATP in the cell culture supernatants were determined with an ENLITIN^®^ ATP Assay System (Promega Corporation, Madison, WI, USA) according to the manufacturer’s instructions.

### Western Blot Analysis

Caco-2 cells (4 × 10^5^ cells/ml) were treated with the indicated stimuli for 3 h. The cell lysates were prepared and Western blotting was performed as described previously (27, 28).

### Determination of DC Migration Using a Trans-Migration Assay

All experiments using human blood were performed after receiving approval from the Institutional Review Board of Seoul National University. Human blood was provided by the Korean Red Cross and all donors provided informed consent, and it was properly handled according to the standard operating procedure for biohazards recommended by the institutional biosafety committee. It has been demonstrated that human blood monocyte-derived DCs upregulated CCR6, which is known to interact with CCL20 (29). To investigate whether CCL20 derived from the co-treatment with Shanchol™ and butyrate, peripheral blood mononuclear cells were isolated by density-gradient centrifugation as described previously (30). For differentiation into immature DCs, purified CD14^+^ monocytes (2 × 10^6^ cells/ml)were cultured in the presence of 10 ng/ml GM-CSF and 10 ng/ml IL-4 for 6 days. Immature DCs (1 × 10^6^ cells/ml) were added to the upper chamber of a 24-well Transwell^®^ support with a 5 µm pore polycarbonate membrane insert (Costar, Corning, NY, USA). Caco-2 cells (4 × 10^5^ cells/ml) were stimulated with Shanchol™ and/or butyrate for 24 h, and then the culture supernatants (600 µl) were collected and moved to the lower chamber of the transwell plate. The cells and supernatants were incubated together at 37°C for 1.5 h. The migrated DCs in the lower chamber were counted using trypan blue staining. For neutralization, culture supernatants were incubated for 30 min at 37°C with 5 µg/ml of anti-CCL20-neutralizing antibody or its isotype control antibody.

### ChIP Assay

Caco-2 cells (4 × 10^5^ cells/ml) were treated with Shanchol™ and/or butyrate for 2 h. The acetylated histone H3 of the CCL20 promoter was determined by using ChIP assay as described previously (31). The cross-linked chromatin DNA was incubated at 4°C overnight with anti-acetylated histone H3 antibodies or its isotype control. The immunoprecipitated DNA was analyzed by real-time RT-PCR using primers specific to the CCL20 promoter (forward 5′-CTT TTC TGG GTC ACA GGG CT-3′ and reverse 5′-GTA CAC AGA AGG CGT GTT GC-3′).

### Transient Transfection and Reporter Gene Assay

Caco-2 cells (5 × 10^5^ cells/ml) were plated for 6 h before transfection. The cells were then transfected overnight with pNF-κB-Luc or pAP-1-Luc (Clontech, Mountain View, CA, USA) using Attractene (Promega). Then, the cells were plated and treated with Shanchol™ and/or butyrate for 16 h. For reporter gene assays, the cells were lysed with Glo Lysis Buffer (Promega) and luciferase activity was then quantified using the Bright-Glo Luciferase Assay System (Promega) with a Spark™ 10-M multimode microplate reader (Tecan Group Ltd., Männedorf, Switzerland).

### Statistical Analysis

All data are expressed as mean value ± SD of triplicates unless otherwise stated. Treatment groups were compared with an appropriate control group. Statistical significance was assessed using ANOVA performed in GraphPad Prism 5 (GraphPad Software Inc., La Jolla, CA, USA). Differences were considered significant when *P* < 0.05.

## Results

### Shanchol™ Induces Chemokine mRNA Expression But Hardly Induces Chemokine Secretion in Human Intestinal Epithelial Cells

**Figure 1:**
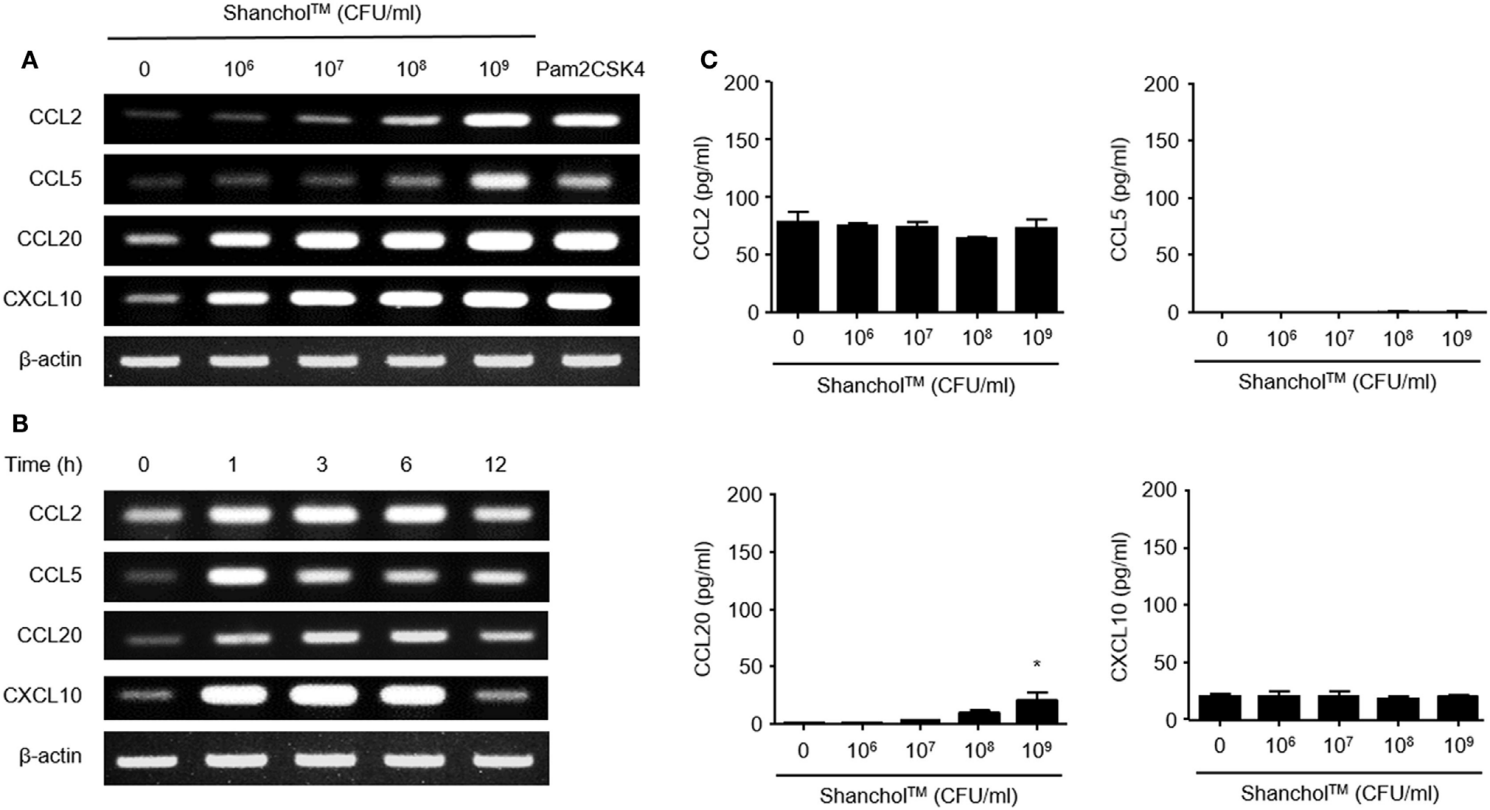
Shanchol™ induces chemokine mRNA expression but hardly induces chemokine secretion in Caco-2 cells. Caco-2 cells were stimulated **(A)** with various concentration of Shanchol™ (10^6^–10^9^ CFU/ml) or Pam2CSK4 (1 µg/ml) for 3 h, or **(B)** with Shanchol™ (10^8^ CFU/ml) for various time periods. Total RNA was extracted and the mRNA expression levels of CCL2, CCL5, CCL20, and CXCL10 were determined by RT-PCR. Data shown are representative of three independent experiments. **(C)** Caco-2 cells were treated with various concentration of Shanchol™ (10^6^–10^9^ CFU/ml) for 24 h. Then, the cell culture supernatants were collected, and the concentrations of CCL2, CCL5, CCL20, and CXCL10 were determined by ELISA. Data shown are representative of three independent experiments. All results are expressed as mean ± SD of triplicate samples. The asterisk (*) indicates a statistically significant difference (*P* < 0.05) compared with control.

First, we examined whether Shanchol™ induces chemokine expression in human intestinal epithelial cells. Shanchol™ treatment (10^6^–10^9^ CFU/ml) induced mRNA expression of CCL2, CCL5, CCL20, and CXCL10 in a dose-dependent manner in Caco-2 cells (Figure 1A and Figure S5 in Supplementary Material). Time course analysis showed that Shanchol™-induced mRNA expression of all chemokines tested peaked at 3–6 h after the treatment, with the exception of CCL5 (Figure 1B and Figure S5 in Supplementary Material). However, the mRNA expression levels of CCL3, CCL4, CCL25, and CCL28 were not altered by Shanchol™ treatment (data not shown). Interestingly, in contrast to the mRNA results, Shanchol™ did not induce secretion of CCL2, CCL5, CCL20, and CXCL10 from Caco-2 cells, though a little induction could be seen at 10^9^ CFU/ml (Figure 1C). Similar results were also observed in HT-29 cells (Figure S1A–C in Supplementary Material). These results indicate that Shanchol™ treatment potently induces mRNA synthesis of chemokines but barely induces protein secretion from human intestinal epithelial cells.

### Co-Treatment of Shanchol™ with Butyrate, but Not with Propionate or Acetate, Potently Induces CCL20 Secretion from Human Intestinal Epithelial Cells

**Figure 2:**
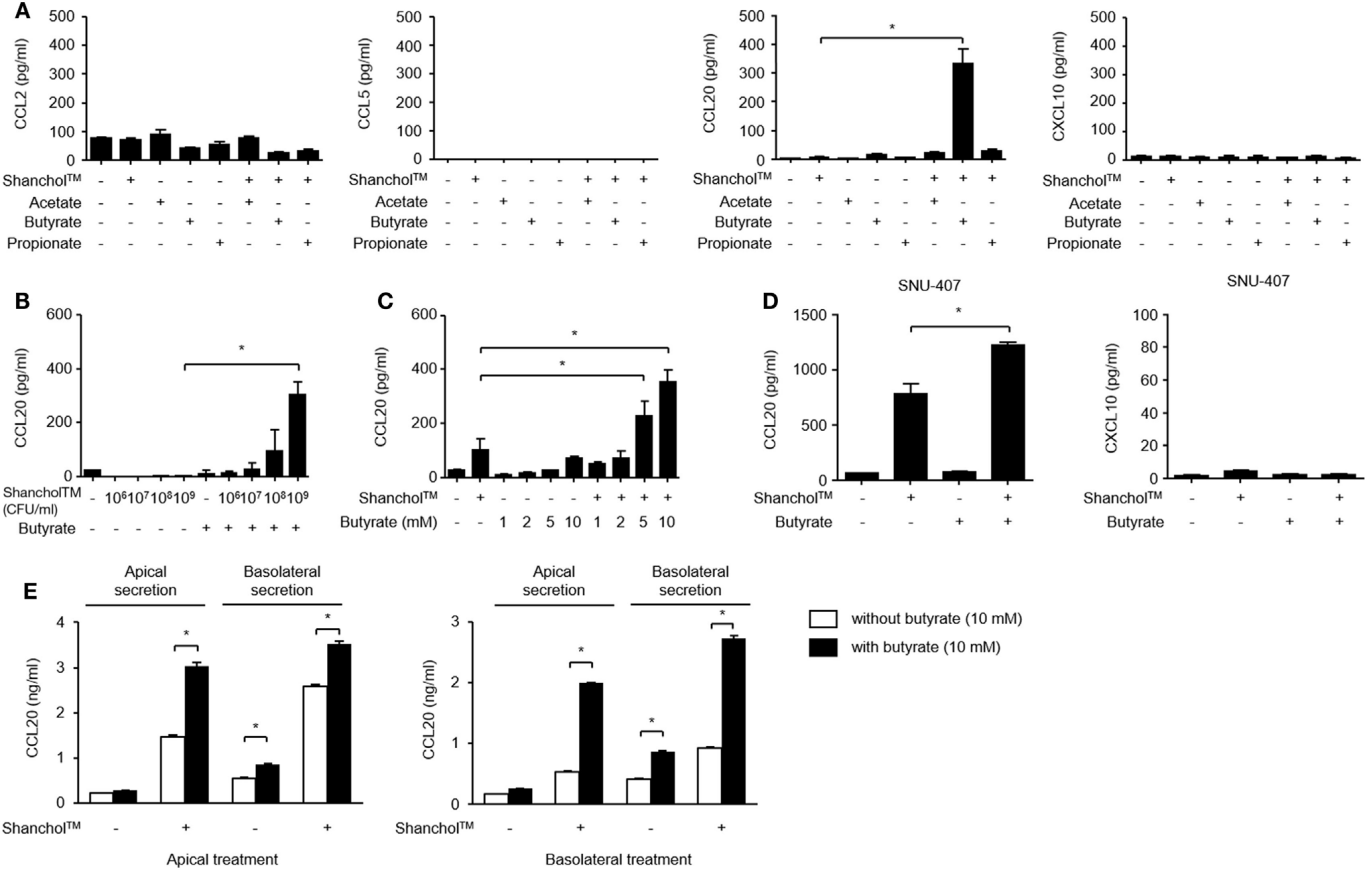
Shanchol™ significantly induces CCL20 secretion in the presence of butyrate in Caco-2 cells. **(A)** Caco-2 cells were stimulated with Shanchol™ (10^9^ CFU/ml) in the presence or absence of acetate, butyrate, or propionate (10 mM). **(B,C)** Caco-2 cells were incubated with various concentrations of Shanchol™ (10^6^–10^9^ CFU/ml) in the presence of butyrate (10 mM) **(B)** or with various concentrations of butyrate (1–10 mM) in the presence of Shanchol™ (10^9^ CFU/ml) **(C)** for 24 h. **(D)** SNU-407 cells were stimulated with Shanchol™ (10^9^ CFU/ml) in the presence or absence of 10 mM butyrate for 24 h. **(E)** Caco-2 cells were plated on Transwell^®^ supports for 4 weeks and then apically or basolaterally treated with Shanchol™ (10^9^ CFU/ml) and/or butyrate (10 mM). The culture supernatants in the apical and basolateral compartments were then collected. The concentrations of secreted CCL2, CCL5, CCL20, and CXCL10 were measured in the culture supernatants by ELISA. N.D., not detected. All results are expressed as mean ± SD of triplicate samples. The asterisk (*) indicates a statistically significant difference (*P* < 0.05) compared with the appropriate control.

Microbiota-associated metabolites such as SCFAs have been reported to play a role in immunomodulation in the gut (32). To examine whether SCFAs alter chemokine secretion, Caco-2 cells were treated with Shanchol™ in the presence of major intestinal SCFAs such as acetate, butyrate, and propionate for 24 h, and chemokine secretion was determined by using ELISA. Interestingly, only butyrate enhanced CCL20 secretion in the presence of Shanchol™. However, neither acetate nor propionate enhanced CCL20 secretion from Caco-2 cells. Remarkably, the secretion of CCL2, CCL5, or CXCL10 was not altered even in the presence of butyrate (Figure 2A). Shanchol™ dose-dependently induced CCL20 secretion in the presence of butyrate (Figure 2B), and butyrate potently increased Shanchol™-induced CCL20 production in a dose-dependent manner (Figure 2C). Such cooperative effect of Shanchol™ and butyrate was observed in the CCL20 mRNA expression, but not in the mRNA expression of other chemokines, including CCL2, CCL5, CXCL10, CCL25, or CCL28 (Figure S2 in Supplementary Material). Furthermore, Shanchol™-induced secretion of CCL20, but not CXCL10, was facilitated by the presence of butyrate in SNU-407 human intestinal epithelial cells (Figure 2D). Caco-2 cells have been reported to differentiate and polarize into cells with intestinal enterocyte-like features (28). Therefore, to mimic the human intestinal epithelium, Caco-2 cells were cultured for 4 weeks on a transwell-permeable filter until the cells were fully differentiated and polarized. As shown in Figure 2E, CCL20 secretion was enhanced in both apical and basolateral compartments of polarized Caco-2 cells, regardless of whether the Shanchol™ and butyrate co-treatment was apical or basolateral.

### GPR109A Is Involved in CCL20 Secretion from Caco-2 Cells

**Figure 3:**
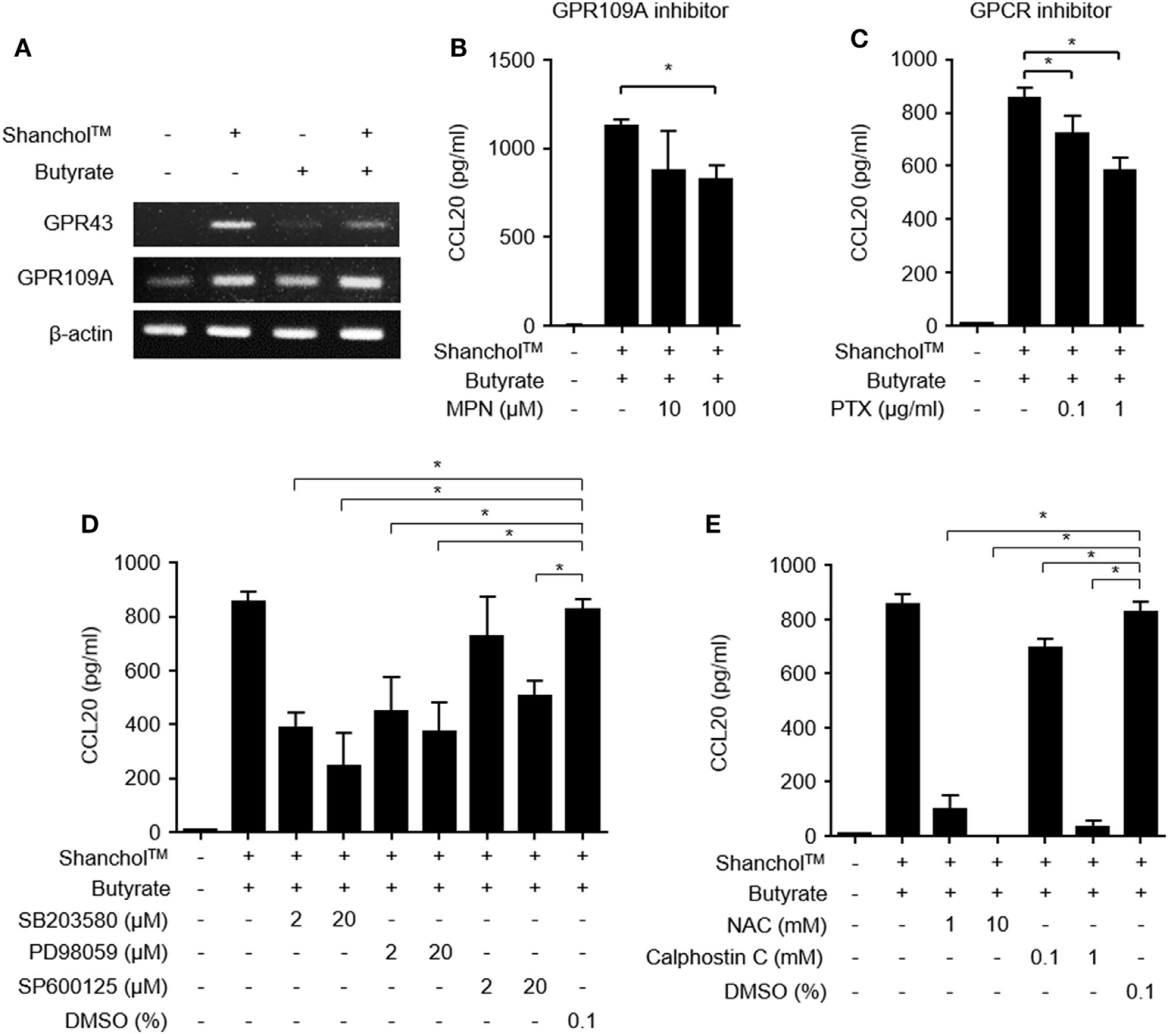
Activation of GPR109A and MAPK is involved in CCL20 secretion from Caco-2 cells. **(A)** Caco-2 cells were stimulated with Shanchol™ (10^9^ CFU/ml) in the presence or absence of butyrate (10 mM) for 3 h. Total RNA was extracted, after which the mRNA expression levels of GPR43 and GPR109A were determined using RT-PCR. **(B–E)** Caco-2 cells were pretreated with the indicated concentrations of mepenzolate bromide (MPN) **(B)**, pertussis toxin (PTX) **(C)**, SB203580, PD98059, SP600125, or DMSO (vehicle control) **(D)**, and NAC, Calphostin C, or DMSO (vehicle control) **(E)** for 1 h. Then, cells were co-stimulated with Shanchol™ (10^9^ CFU/ml) and butyrate (10 mM) for an additional 24 h, the culture supernatants were collected, and the concentrations of secreted CCL20 were measured by ELISA. One of three representative results is shown. All results are expressed as mean ± SD of triplicate samples. The asterisk (*) indicates a statistically significant difference (*P* < 0.05) compared with the appropriate control.

GPR43 and GPR109A are butyrate receptors that regulate various immune responses such as leukocyte migration and lymphocyte activation (33). It has been reported that Gram-negative bacteria and their lipopolysaccharide (LPS) induced GPR43 and GPR109A expression (34, 35). To determine whether GPR43 or GPR109A is involved in butyrate-mediated stimulation of CCL20 secretion, we examined GPR43 and GPR109A expression in Caco-2 cells. In Caco-2 cells treated with Shanchol™, mRNA expression of both GPR43 and GPR109A was induced and butyrate augmented only GPR109A mRNA expression but not GPR43 mRNA expression (Figure 3A and Figure S5 in Supplementary Material). We next examined whether GPR109A is involved in CCL20 secretion. When Caco-2 cells were pretreated with a specific inhibitor of GPR109A (MPN) or a GPCR inhibitor (pertussis toxin) for 1 h and then treated with Shanchol™ and/or butyrate for an additional 24 h, CCL20 secretion was significantly suppressed by both GPR109A inhibitor and GPCR inhibitor (Figures 3B,C). Since the activation of GPR109A is associated with the downstream signaling mediators, such as mitogen-activated protein kinase (MAPK), protein kinase C (PKC), and reactive oxygen species (ROS) (36–38), we investigated the enhancement of CCL20 production in Caco-2 cells pretreated with the specific inhibitors for MAPK, PKC, or ROS. CCL20 secretion was remarkably inhibited by MAPK-specific inhibitors (SB203580 for p38 kinase, PD98059 for ERK, and SP600125 for JNK) (Figure 3D). Furthermore, CCL20 secretion was downregulated by pretreatment with inhibitors for ROS or PKC (Figure 3E), suggesting that GPR109A, MAPK, PKC, and ROS signaling mediate the enhancement of CCL20 secretion in Caco-2 cells.

### ATP-P2X7 Signaling Is Required for CCL20 Secretion by Caco-2 Cells

**Figure 4:**
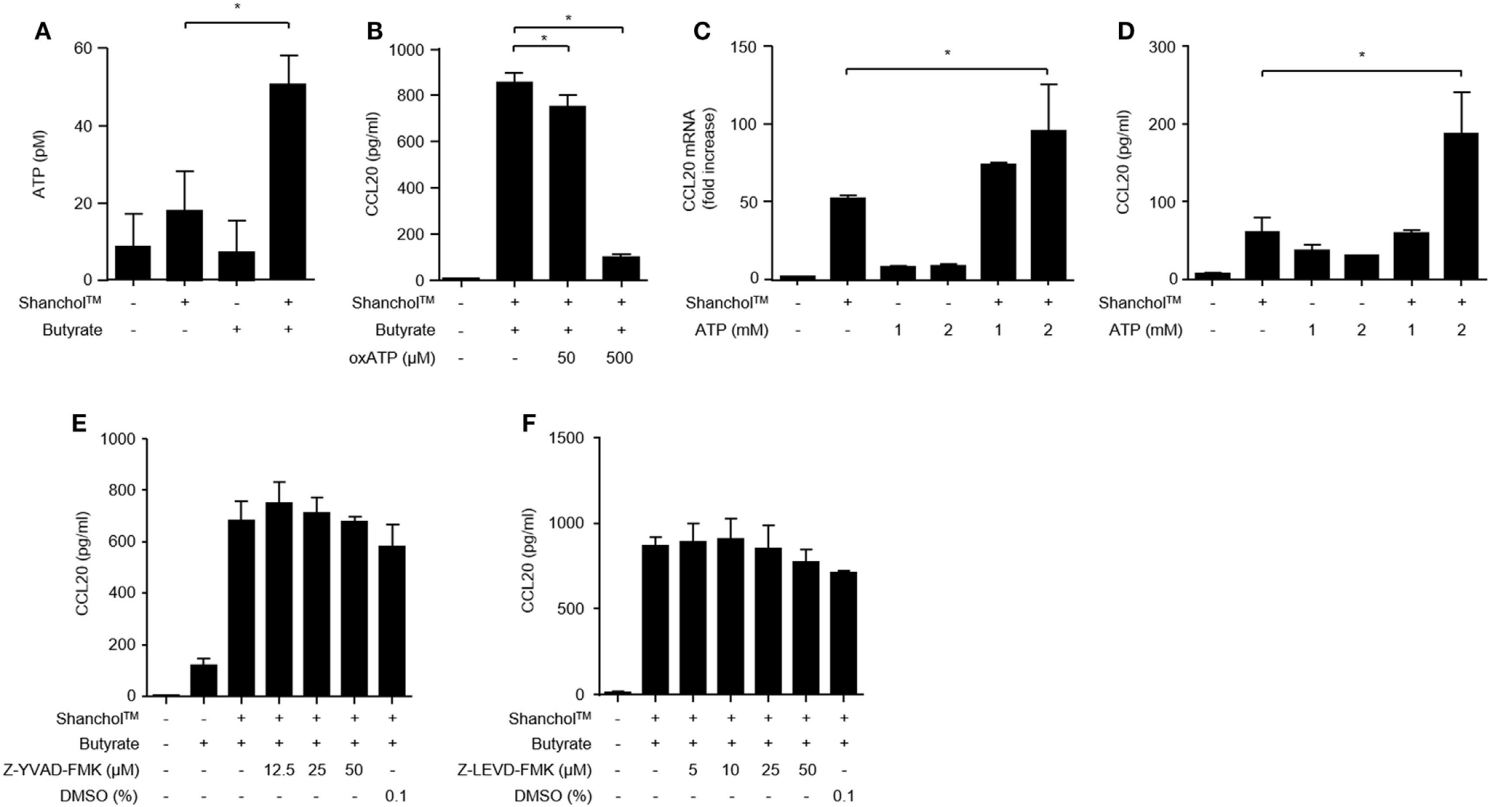
ATP is required for CCL20 secretion from Caco-2 cells. **(A)** Caco-2 cells were incubated with Shanchol™ in the presence or absence of 10 mM butyrate for 2 h. Then, the culture supernatants were collected, and extracellular ATP production was measured. **(B)** Caco-2 cells were pretreated with 50 or 500 µM of oxATP for 1 h and subsequently stimulated with Shanchol™ and butyrate for an additional 24 h. Then, the culture supernatants were collected, and the concentrations of secreted CCL20 were measured by ELISA. Data shown are representative of three independent experiments. **(C)** Caco-2 cells were stimulated with the indicated concentration of ATP in the presence or absence of Shanchol™ for 3 h. Total RNA was isolated, and the mRNA expression level of CCL20 was determined by real-time RT-PCR. **(D)** Caco-2 cells were stimulated with the indicated concentration of ATP in the presence or absence of Shanchol™ for 24 h. Then, the culture supernatants were collected, and the secreted CCL20 was measured by ELISA. **(E,F)** Caco-2 cells were pretreated with the indicated concentrations of the caspase-1 inhibitor (Z-YVAD-FMK) **(E)**, the caspase-4 inhibitor (Z-LEVD-FMK) **(F)**, or DMSO (vehicle control) for 1 h and subsequently stimulated with Shanchol™ and butyrate for an additional 24 h. Then, the culture supernatants were collected and the CCL20 secretion was measured by ELISA. Data shown are representative of three independent experiments. All results are expressed as mean ± SD of triplicate samples. The asterisk (*) indicates a statistically significant difference (*P* < 0.05) compared with the appropriate control.

Butyrate is a source of metabolic energy (i.e., ATP) in colonocytes (39), where ATP enhances IL-6 and KC chemokine production in murine intestinal epithelial cells (40). To investigate whether butyrate induces ATP secretion in Caco-2 cells, the extracellular concentrations of ATP were determined in the presence or absence of the Shanchol™/butyrate. As shown in Figure 4A, extracellular ATP production was increased in Caco-2 cells co-treated with Shanchol™ and butyrate. Blocking the interaction between ATP and P2X7 receptor by treatment with a P2X7 purinergic receptor antagonist (oxATP) dramatically attenuated CCL20 secretion (Figure 4B). Moreover, exogenously treated ATP augmented CCL20 mRNA expression and secretion in the presence of Shanchol™ (Figures 4C,D, respectively), whereas addition of exogenous ATP did not affect Shanchol™-induced CXCL10 mRNA expression or secretion (Figure S3 in Supplementary Material). These results suggest that butyrate-induced ATP secretion is crucial for the enhancement of Shanchol™-induced CCL20 production. However, pretreatment with a caspase-1 or caspase-4 inhibitor did not suppress CCL20 secretion (Figures 4E,F, respectively). Thus, those results suggest that ATP-P2X7 signaling, but not inflammasome activation, is essential for the synergistic production of CCL20 in response to Shanchol^TM^ and butyrate.

### HDAC Inhibition Is Associated with Enhanced CCL20 Production in Caco-2 Cells

**Figure 5:**
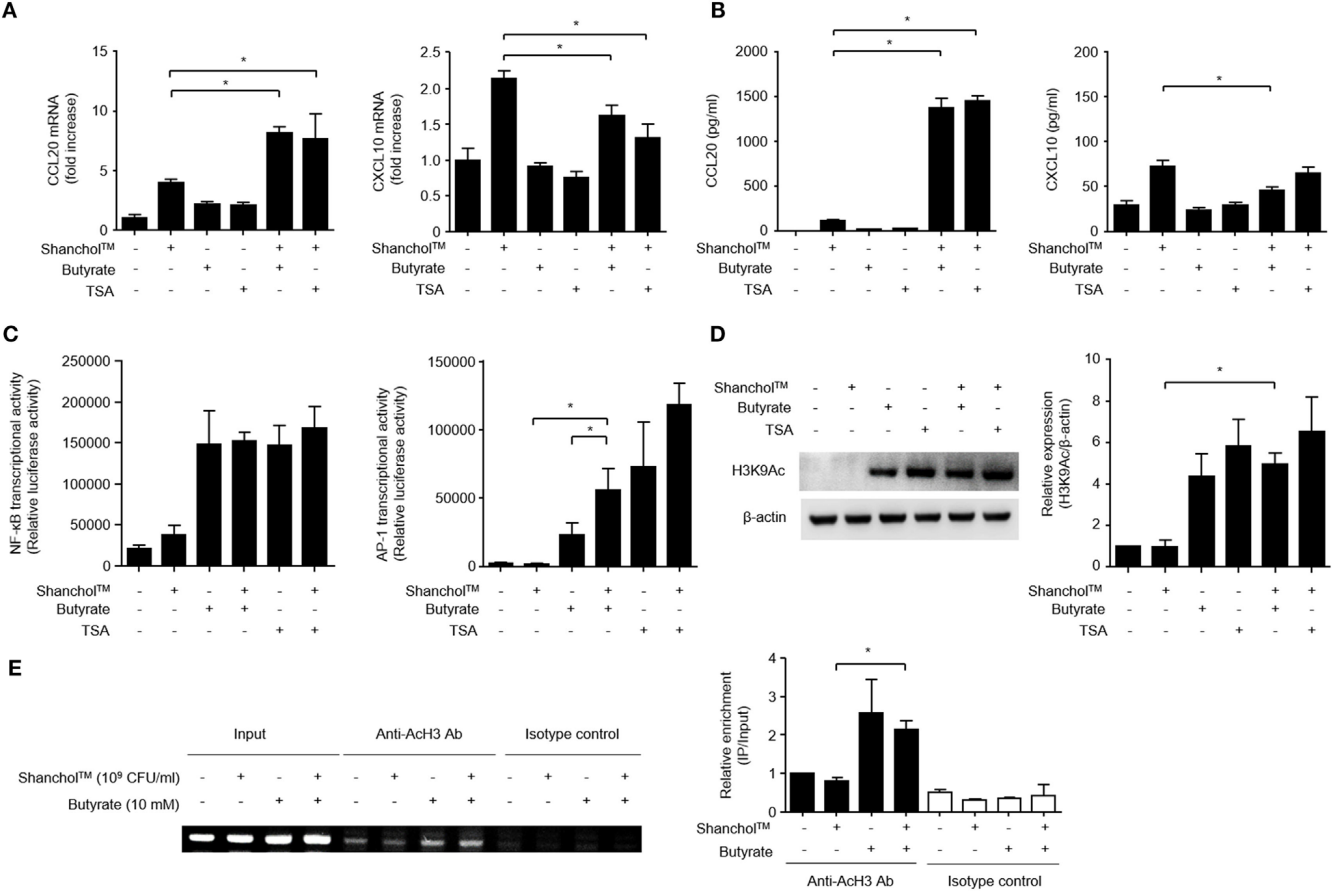
Histone deacetylase inhibition is necessary for CCL20 production in Caco-2 cells. **(A)** Caco-2 cells were stimulated with Shanchol™ in the presence of 10 mM butyrate or 5 µM trichostatin A (TSA) for 3 h. Total RNA was isolated, and the mRNA expression level of CCL20 or CXCL10 was determined by real-time RT-PCR. **(B)** Caco-2 cells were treated with Shanchol™ in the presence of 10 mM butyrate or 5 µM TSA for 24 h. Then, the culture supernatants were collected, and the concentrations of CCL20 and CXCL10 were determined by ELISA. All results are expressed as mean ± SD of triplicate samples. **(C)** Caco-2 cells transfected with a pNF-κB-luc or pAP-1uc luciferase plasmid were further treated with Shanchol™ in the presence of butyrate or TSA for 16 h. Then, the cells were lysed, and subjected to the luciferase assay. **(D)** Caco-2 cells were stimulated with Shanchol™ in the presence of butyrate or TSA for 3 h. Cell lysates were generated and subjected to Western blot analysis to examine histone acetylation at lysine residues. The relative ratio of acetylated-H3K9 to β-actin was obtained by densitometry. **(E)** Caco-2 cells were treated with Shanchol™ and/or butyrate for 2 h. Then, the cells were subjected to ChIP assay. Cross-linked chromatin extracts were immunoprecipitated with anti-acetylated histone H3 antibodies (Anti-AcH3 Ab) or rabbit IgG antibodies (Isotype control). DNA fragments were analyzed by PCR (*left*) and by real-time RT-PCR (*right*) using primers specific to the human CCL20 promoter. The results shown are representative of three independent experiments. Data are presented as mean ± SD of three independent experiments. The asterisk (*) indicates a statistically significant difference (*P* < 0.05) compared with the appropriate control.

Butyrate has been reported to act as an HDAC inhibitor (41) and regulates the transcription of chemokines and antimicrobial peptides in epithelial cells (42). Therefore, Caco-2 cells were treated with Shanchol™ in the presence of TSA, an HDAC inhibitor. CCL20 mRNA expression was increased by co-treatment with Shanchol™ and TSA, similar to the results for co-treatment with Shanchol™ and butyrate. However, TSA did not enhance Shanchol™-induced CXCL10 mRNA expression as butyrate did not (Figure 5A). Besides, co-treatment with Shanchol™ plus butyrate or Shanchol™ plus TSA resulted in increased CCL20 secretion, but not increased CXCL10 secretion (Figure 5B). The CCL20 promoter region contains binding sites for the transcription factors NF-κB and AP-1. Reporter gene assay showed that co-treatment with Shanchol™ and butyrate synergistically induced AP-1 activation, while butyrate alone or together with Shanchol™ substantially increased NF-κB activation (Figure 5C). Increased acetylation of histone H3 lysine residues was observed when cells were co-treated with butyrate or TSA (Figure 5D and Figure S6 in Supplementary Material). ChIP assay showed that butyrate augmented acetylation of histone H3 in the CCL20 promoter region though co-treatment with Shanchol™ and butyrate did not enhance histone acetylation (Figure 5E and Figure S7 in Supplementary Material). Taken together, these results suggest that HDAC inhibition by butyrate is involved in synergistic CCL20 production at the transcriptional level in Caco-2 cells.

### Co-Treatment with Shanchol™ and Butyrate Induces Chemotactic Migration of Human Immature DCs

**Figure 6:**
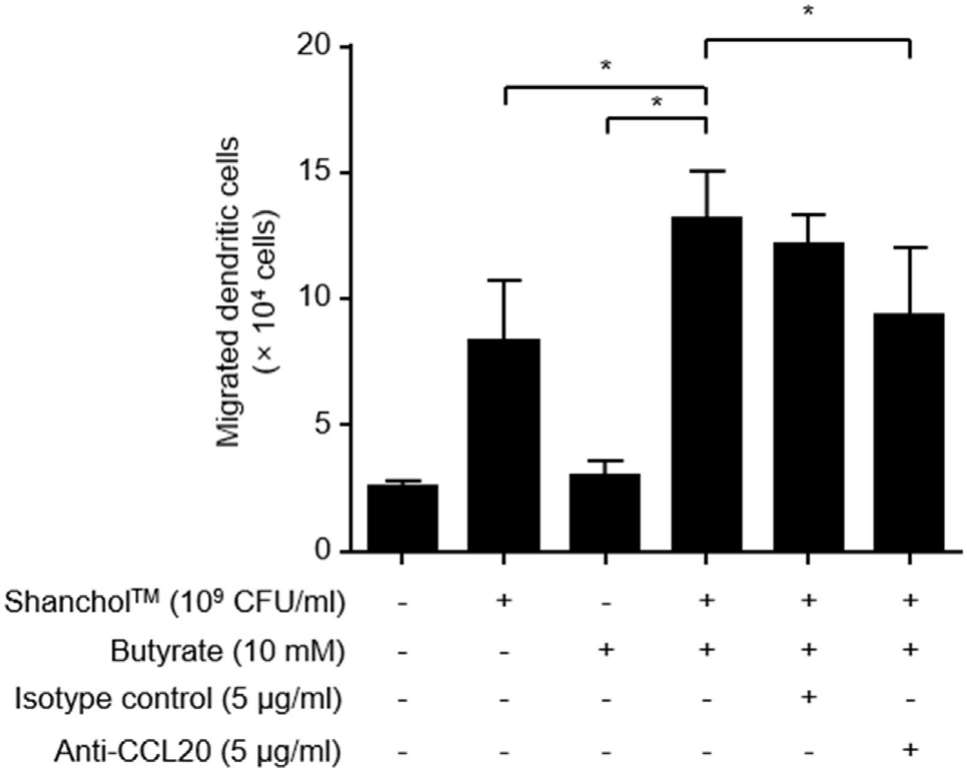
Co-treatment with Shanchol™ and butyrate induces chemotactic migration of human immature dendritic cells (DCs). Caco-2 cells were stimulated with Shanchol™ (10^9^ CFU/ml) in the presence or absence of butyrate (10 mM) for 24 h. After that, the culture supernatants were pre-incubated with indicated concentration of anti-CCL20-neutralizing antibody or isotype control for 30 min. Immature DCs were placed in the upper chamber and further incubated for 1.5 h. The migrated DCs in the lower chamber were counted using trypan blue staining. All results are expressed as mean ± SD of triplicate samples. The asterisk (*) indicates a statistically significant difference (*P* < 0.05) compared with the appropriate control.

CCL20 has been shown to promote the migration of immature DCs (43). Thus, we assessed the migratory capacity of human immature DCs in response to Shanchol™ and butyrate. Human immature DCs expressed CCR6 which is the receptor for CCL20 (Figure S4 in Supplementary Material). Significantly more DC migration was observed in response to co-treatment with Shanchol™ and butyrate compared with the level in response to either Shanchol™ or butyrate alone (Figure 6), suggesting that co-treatment with Shanchol™ and butyrate-induced CCL20 might be associated with the augmented DC migration. In addition, the migration of DCs was inhibited by incubation with CCL20-neutralizing antibodies indicating that this migration specifically depends on CCL20-CCR6 chemotaxis.

## Discussion

**Figure 7:**
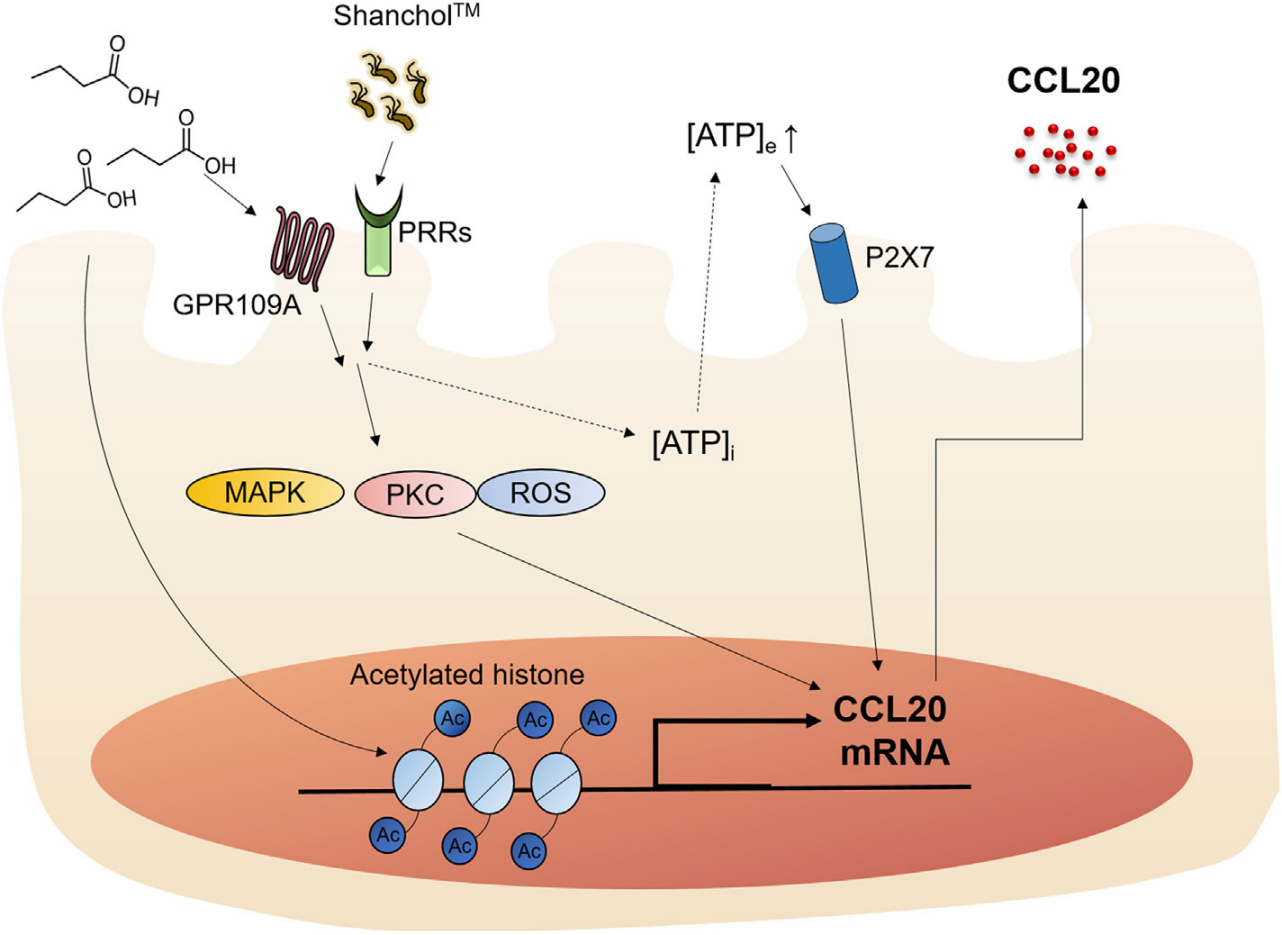
Schematic illustration of the proposed action mechanism. Butyrate enhances Shanchol™-induced CCL20 secretion *via* GPR109A, ATP-P2X7, and epigenetic control by histone deacetylase inhibition.

SCFAs are known to modulate the host immune responses by binding to receptors such as GPR43 and GPR109A or through HDAC inhibition (19). Butyrate is an SCFA that has been shown to stimulate GPR109A and enhance IL-18 production in the intestinal epithelium (44). However, the mechanisms by which SCFAs affect vaccine-induced chemokine expression in intestinal epithelial cells have not yet been elucidated. In the present study, we showed that the killed whole-cell oral cholera vaccine Shanchol™ potently induced chemokine mRNA expression in human intestinal epithelial cells, whereas it barely induced chemokine protein secretion. Consistent with our results, IL-8 mRNA expression has been shown to be substantially induced in HT-29-18N2 cells in response to cholera vaccine strains, whereas negligible protein secretion was observed (<50 pg/ml) (45). Notably, CCL20 was reported to be expressed at both mRNA and protein levels in follicle-associated epithelium of Peyer’s patches but not in the intestinal villus epithelium in mice (46). This discrepancy might be attributed to species difference, because CCL20 was weakly expressed in normal human colon epithelium and its expression was substantially increased in response to inflammatory cytokines (47). In addition, the present study demonstrated that butyrate potently enhanced Shanchol™-induced expression of CCL20 in human intestinal epithelial cells, but not any other chemokines tested. Our studies and others for elucidating the relevant molecular mechanisms have demonstrated that epigenetic modification (48), ATP-P2X7 signaling (49) and GPR109A-mediated pathways are associated with butyrate-mediated enhancement of CCL20 production, as summarized in Figure 7. We also showed that migration of human immature DCs was increased in the presence of Shanchol™ and butyrate, implying that butyrate contributes to the enhancement of mucosal immune responses induced by oral cholera vaccines in the gut.

Gram-negative bacteria contain diverse microbe-associated molecular patterns, such as LPS and lipoproteins, which are generally recognized by toll-like receptor 4 (TLR4) and TLR2, respectively (50). Although Shanchol™ comprises Gram-negative bacteria, *V. cholerae* strains, our group showed that *V. cholerae* preferentially induced TLR2 activation, but not TLR4 activation, ultimately resulting in pro-inflammatory responses (51). Furthermore, a porin protein of *V. cholerae*, OmpU, exclusively interacted with TLR2, and induced IL-8 production (52), while LPS of *V. cholerae* failed to induce IL-8 production in HT-29 cells (9). Although further comprehensive studies are needed, OmpU of *V. cholerae* would be one of the major components to induce chemokine production in human intestinal epithelial cells.

SCFAs are saturated fatty acids consisting of one to six carbons; acetate (C2), propionate (C3), and butyrate (C4). The carbon chain length of SCFAs has been proposed to affect their immunomodulatory potencies (53). In support of this idea, acetate, propionate, and butyrate all activate GPCRs, whereas GPR109A is recognized only by butyrate but not by acetate or propionate (18). In addition, chain length of SCFAs affects the ability to stimulate chemokine production by altering histone acetylation. SCFAs with longer carbon chain lengths are known to be more potent inducers of histone acetylation (53). For example, butyrate has more potent histone-acetylating activity by interfering with HDAC compared with acetate and propionate (42). In this study, we demonstrated that only butyrate substantially augments CCL20 production in Caco-2 cells. Furthermore, we also found that the GPR109A-mediated pathways and HDAC inhibition are associated with enhancement of CCL20 production by butyrate. Thus, the chain length of SCFAs might be associated with the potency of the SCFA to activate GPCRs and regulate epigenetic modification, consequently resulting in the modulation of chemokine expression by butyrate.

Increased level of intracellular ATP was found in Caco-2 cells in response to butyrate (54). However, bacterial components including TLR ligands promoted extracellular ATP production (55). Indeed, we found that simultaneous treatment with Shanchol™ and butyrate significantly increased extracellular ATP production, while butyrate alone could not increase extracellular ATP level. Furthermore, supernatants from butyrate-treated Caco-2 cells without Shanchol™ treatment did not considerably induce CCL20 production (Figure S8 in Supplementary Material), suggesting that Shanchol™ stimulates the excretion of ATP to induce CCL20 production.

In this study, we demonstrated that ATP-P2X7 signaling plays a key role in CCL20 production in Caco-2 cells. This finding is consistent with previous studies reporting that high levels of ATP were released to the extracellular space, and that purinergic receptors such as P2X7 were activated in epithelial cells upon exposure to mechanical stress, hypotonic media, vasoactive agents, or inflammatory stimuli (56). This release of ATP resulted in the release of CCL2, CCL5, and CXCL8, while it inhibited the expression of CXCL10 *via* P2X7 and P2Y1 receptor signaling (57). Activation of the P2X7 receptor by extracellular ATP induces Ca^2+^ influx and K^+^ efflux, thereby activating the NLRP3 inflammasome (58). However, pretreatment with caspase-1 or caspase-4 inhibitors did not affect CCL20 production in this study, suggesting that inflammasome activation is not involved in CCL20 secretion. In addition to activating the inflammasome, P2X7 receptor activation increases the intracellular Ca^2+^ concentration, thereby activating downstream signaling pathways such as the MAPK p38 pathway (59). However, our observation indicates that an intracellular Ca^2+^ chelating regent, BAPTA-AM, did not abrogate CCL20 production (Figure S9 in Supplementary Material), suggesting that Ca^2+^ influx is not involved in the enhancement of CCL20 secretion. Although further studies are needed, butyrate-mediated GPR109A and ATP-P2X7 may induce intracellular signaling pathways such as MAPK pathway, leading to the enhancement of CCL20 secretion.

Epigenetic control is a phenomenon by which gene expression and function are regulated in various cell types without changing the nucleotide sequence. This type of control is mediated by histone modification and DNA methylation. Histone acetylation is catalyzed by histone acetyltransferases, and is related to transcriptional activation by allowing the transcription machinery to access the DNA binding sites. We found that HDAC inhibition by butyrate or TSA induced histone acetylation at lysine residues and strongly increased Shanchol™-induced CCL20 mRNA and protein production in Caco-2 cells. In accordance with our observation, butyrate has been shown to induce CCL20 production on the transcriptional level *via* HDAC inhibition in gingival epithelial cells (48) and in intestinal epithelial cells (60). However, we also found that butyrate and TSA synergistically increased AP-1 and downregulated the expression of other chemokines (e.g., CCL2 and CXCL10). These findings are consistent with previous reports that butyrate or TSA enhances PMA-induced AP-1 response in human intestinal cells (61), and AP-1 negatively regulated CXCL10 induction in response to TLR3 or RIG-1 ligand in hepatocytes (62). In addition, it has been demonstrated that HDAC inhibition enhances TLR-induced TNF-α expression in HT-29 cells, whereas it blocks IL-8 and MCP-1 expression (63). Therefore, it is likely that HDAC inhibition alone cannot upregulate the expression of all chemokines on the transcriptional level.

Chemokine production is important for the induction of mucosal immune responses following vaccination. Although many chemokines are associated with inflammatory responses, recent researches in the biological roles of chemokines such as CCL20 also allow to employ as potential vaccine adjuvants by regulating mucosal immune responses (16, 43). Studies have shown that mucosal vaccination increases local chemokine production in the gut mucosa; these chemokines attract immune cells (including IgA-producing plasma cells) to the site of vaccination (64, 65). In fact, chemokines such as CCL20 have been used as adjuvants for several cancer vaccines and for DNA vaccines against simian immunodeficiency virus infection (66–68). CCR6, the CCL20 receptor, is important for the recruitment of DCs, memory T cells, and B cells, which are responsible for protective immunity. Several studies have shown that gut microbiota is closely associated with vaccine effectiveness (69, 70). The treatment with prebiotics such as dietary fibers promotes the production of SCFAs in the gut that increases the immunogenicity and efficacy of vaccines (71, 72). In addition, the administration with *Bifidobacterium*, which is a major bacterium producing SCFAs in the intestine, evidently increased the level of serum and fecal IgA (73), suggesting that the SCFA such as butyrate could enhance the effectiveness of cholera vaccine. We found that co-treatment with Shanchol™ and butyrate potently induced CCL20 secretion. Since this cytokine is associated with the migration of immature DCs, our results suggest that butyrate accelerates Shanchol™-induced mucosal immune responses in the gut. Since CCL20 induces chemotaxis of immune cells that impact vaccine efficacy, our findings indicate that butyrate could be used to enhance mucosal vaccine-induced immune responses. Although further studies are needed to establish that the use of butyrate correlates with the induction of protective immunity, the finding that butyrate regulates intestinal immune responses when used together with an oral cholera vaccine provides an important first step toward achieving enhanced mucosal vaccine efficacy in the gut.

## Ethics Statement

This study was carried out in accordance with the recommendations of Institutional Review Board with written informed consent from all subjects and with the Declaration of Helsinki.

## Author Contributions

SH conceived the idea. J-RS, S-SK, and SH designed the experiments. J-RS and S-SK performed experiments. J-RS, S-SK, and SH analyzed and/or interpreted the data and contributed to the discussion of the results followed by writing and reviewing the manuscript. DL and C-HY provided critical comments and contributed to the discussion of the results followed by writing and reviewing the manuscript.

## Conflict of Interest Statement

The authors declare that the research was conducted in the absence of any commercial or financial relationships that could be construed as a potential conflict of interest.

## Abbreviations

CTB, cholera toxin B subunit; GALT, gut-associated lymphoid tissue; APC, antigen-presenting cell; DC, dendritic cell; SCFA, short-chain fatty acid; HDAC, histone deacetylase; GPCR, G-protein-coupled receptor; ATP, adenosine triphosphate; TSA, trichostatin A; MPN, mepenzolate bromide; PTX, pertussis toxin; ROS, reactive oxygen species; PKC, protein kinase C; HAT, histone acetyltransferase.
