# Supplementary material for: Killed Whole-Cell Oral Cholera Vaccine Induces CCL20 Secretion by Human Intestinal Epithelial Cells in the Presence of the Short-Chain Fatty Acid, Butyrate
Source: Front Immunol. 2018 Jan 29;9:55. doi: 10.3389/fimmu.2018.00055 (PMC5796904; doi:10.3389/fimmu.2018.00055)
Supplement: Supplementary file 1 [file data_sheet_1.docx]

Supplementary Material

Killed whole-cell oral cholera vaccine induces CCL20 secretion by human intestinal epithelial cells in the presence of the short chain fatty acid, butyrate

**Ju-Ri Sim, Seok-Seong Kang, Daesang Lee, Cheol-Heui Yun and Seung Hyun Han***

*** Correspondence:** Seung Hyun Han: [shhan-mi@snu.ac.kr](mailto:shhan-mi@snu.ac.kr)


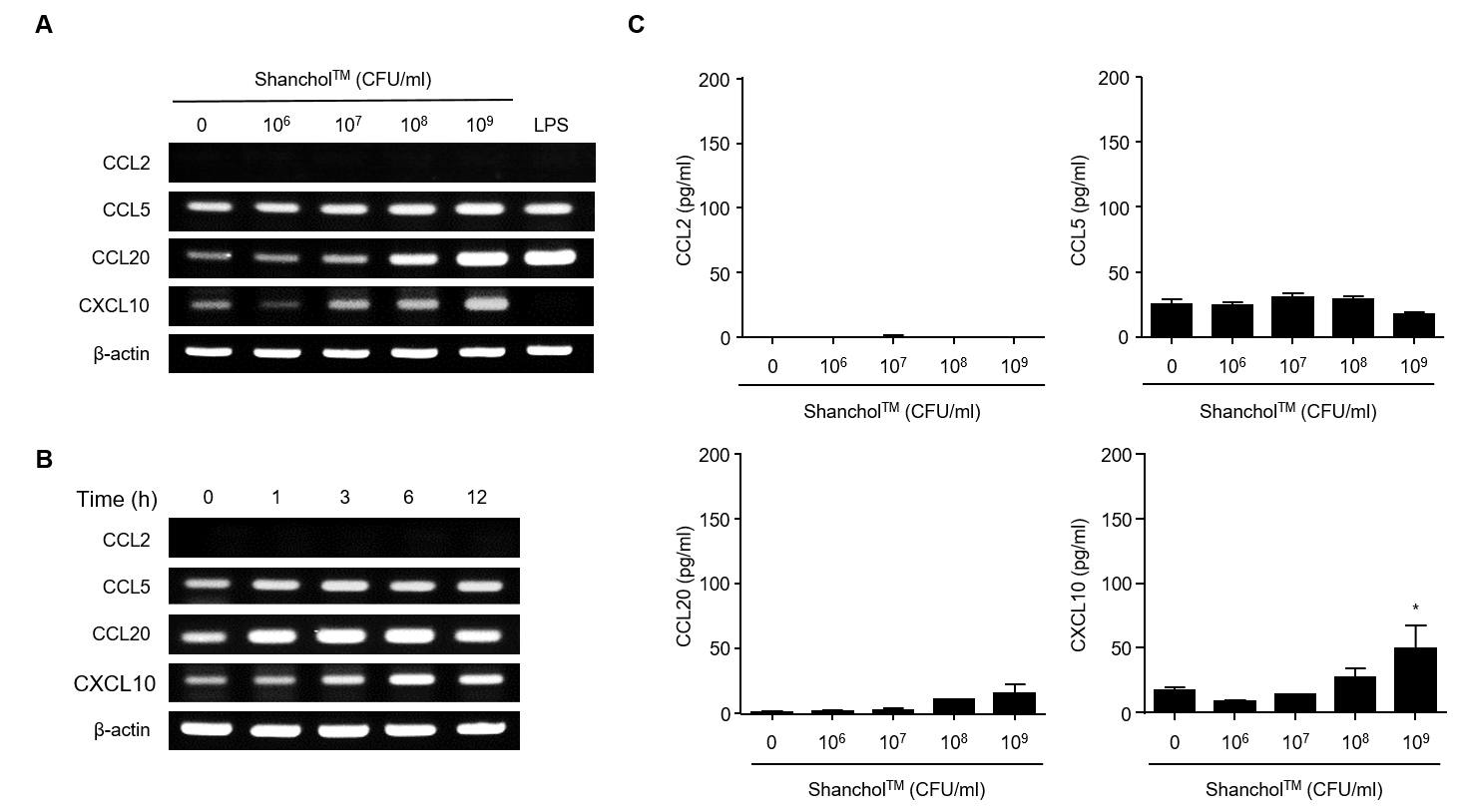


**Supplementary Figure 1** **Shanchol™ induces chemokine mRNA expression but hardly induces chemokine secretion in HT-29 cells.**

HT-29 cells were stimulated (A) with various concentration of Shanchol^TM^ (10^6^ to 10^9^ CFU/ml) or LPS (1 μg/ml) for 3 h, or (B) with Shanchol^TM^ (10^8^ CFU/ml) for various time periods. Total RNA was extracted and the mRNA expression levels of CCL2, CCL5, CCL20, and CXCL10 were determined by RT-PCR. Data shown are representative of three independent experiments. (C) HT-29 cells were treated with various concentration of Shanchol^TM^ (10^6^ to 10^9^ CFU/ml) for 24 h. Then, the cell culture supernatants were collected, and the concentrations of CCL2, CCL5, CCL20, and CXCL10 were determined by ELISA. Data shown are representative of three independent experiments. All results are expressed as mean ± SD of triplicate samples. The asterisk (*) indicates a statistically significant difference (*P* < 0.05) compared with control.


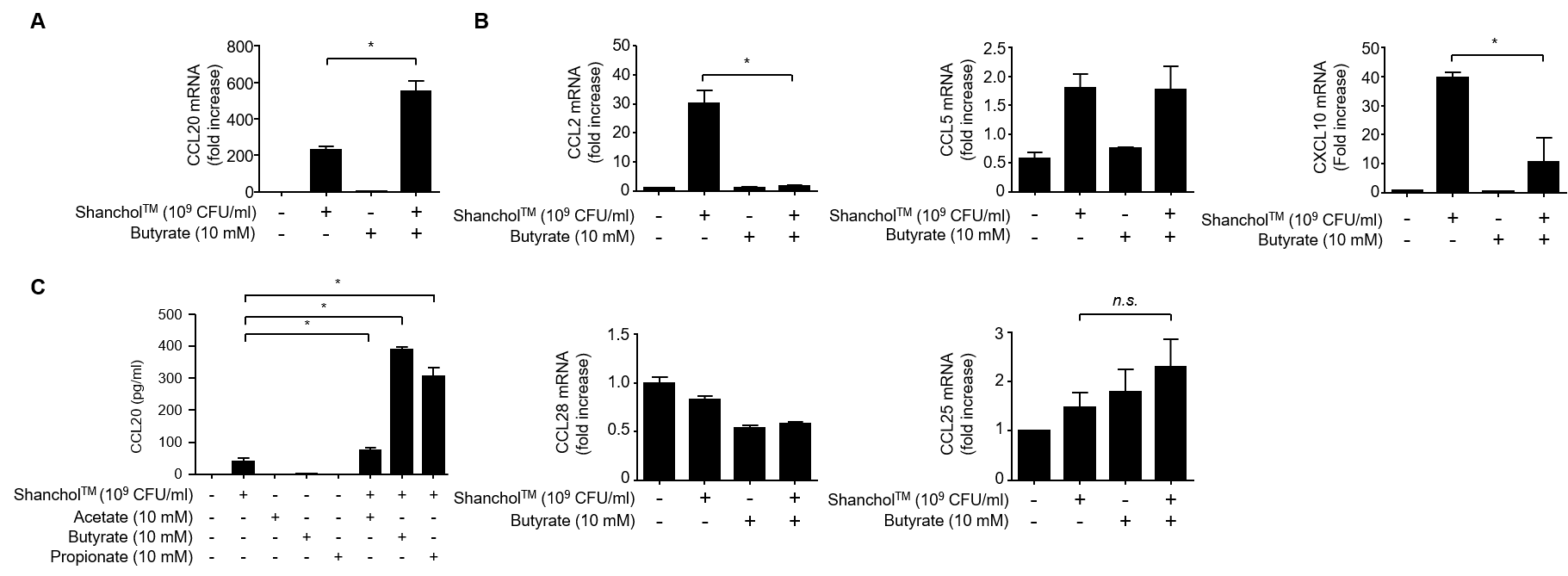


**Supplementary Figure 2 mRNA expression of CCL20 is up-regulated by co-treatment with Shanchol^TM^ and butyrate.**

(A and B) Caco-2 cells were stimulated with Shanchol^TM^ (10^9^ CFU/ml) in the presence or absence of butyrate (10 mM) for 3 h. Total RNA was extracted and the mRNA expression level of CCL2, CCL5, CXCL10, CCL20 and CCL28 were determined by real-time PCR. CCL25 mRNA expression was determined by RT-PCR followed by normalization to β-actin after densitometric analysis. Data are presented as mean ± SD of three independent experiments. (C) HT-29 cells were stimulated with Shanchol^TM^ (10^9^ CFU/ml) in the presence or absence of acetate, butyrate, or propionate at 10 mM. The culture supernatants were collected, and the concentrations of secreted CCL20 were measured by ELISA. All results are expressed as mean ± SD of triplicate samples. The asterisk (*) indicates a statistically significant difference (*P* < 0.05) compared with the appropriate control. *n.s.* indicates not significant.


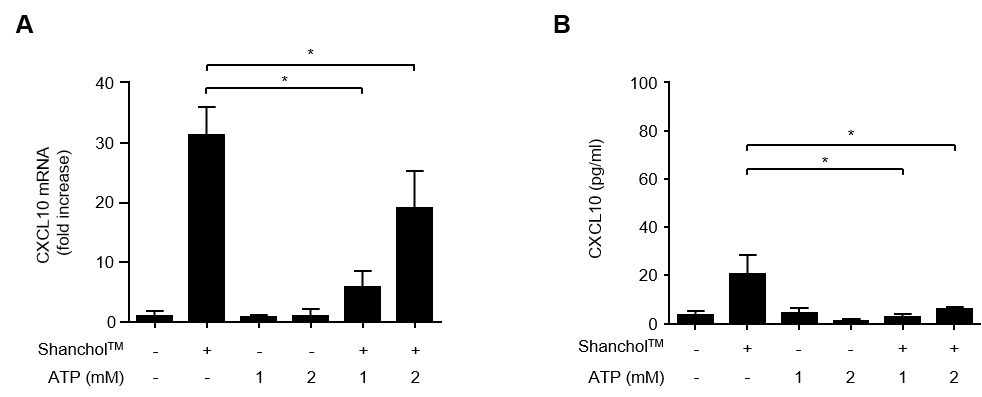


**Supplementary Figure 3 Addition of exogenous ATP does not enhance Shanchol™-induced CXCL10 mRNA expression or its protein secretion.**

**(A)** Caco-2 cells were stimulated with the indicated concentration of ATP in the presence or absence of Shanchol^TM^ (10^9^ CFU/ml) for 3 h. Total RNA was isolated, and the mRNA expression level of CXCL10 was determined by real-time PCR. **(B)** Caco-2 cells were stimulated with the indicated concentration of ATP in the presence or absence of Shanchol^TM^ (10^9^ CFU/ml) for 24 h. Then, the culture supernatants were collected, and the secreted CXCL10 was measured by ELISA. All results are expressed as mean ± SD of triplicate samples. The asterisk (*) indicates a statistically significant difference (*P* < 0.05) compared with the appropriate control.


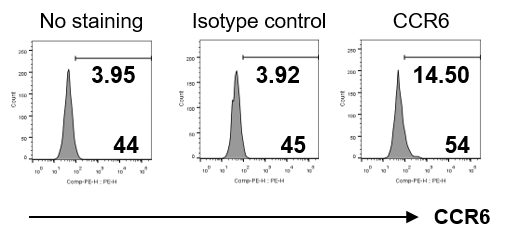


**Supplementary Figure 4 Expression of CCR6 on human immature DCs.**

Human CD14^+^ monocytes were cultured for 6 days in the presence of GM-CSF and IL-4 to differentiate into immature DCs. Expression of CCR6 on immature DCs without treatment was analyzed by flow cytometry. The numbers on the histogram indicate the percentage (*upper*) or the mean fluorescence intensity (MFI) (*lower*) of DCs.

**
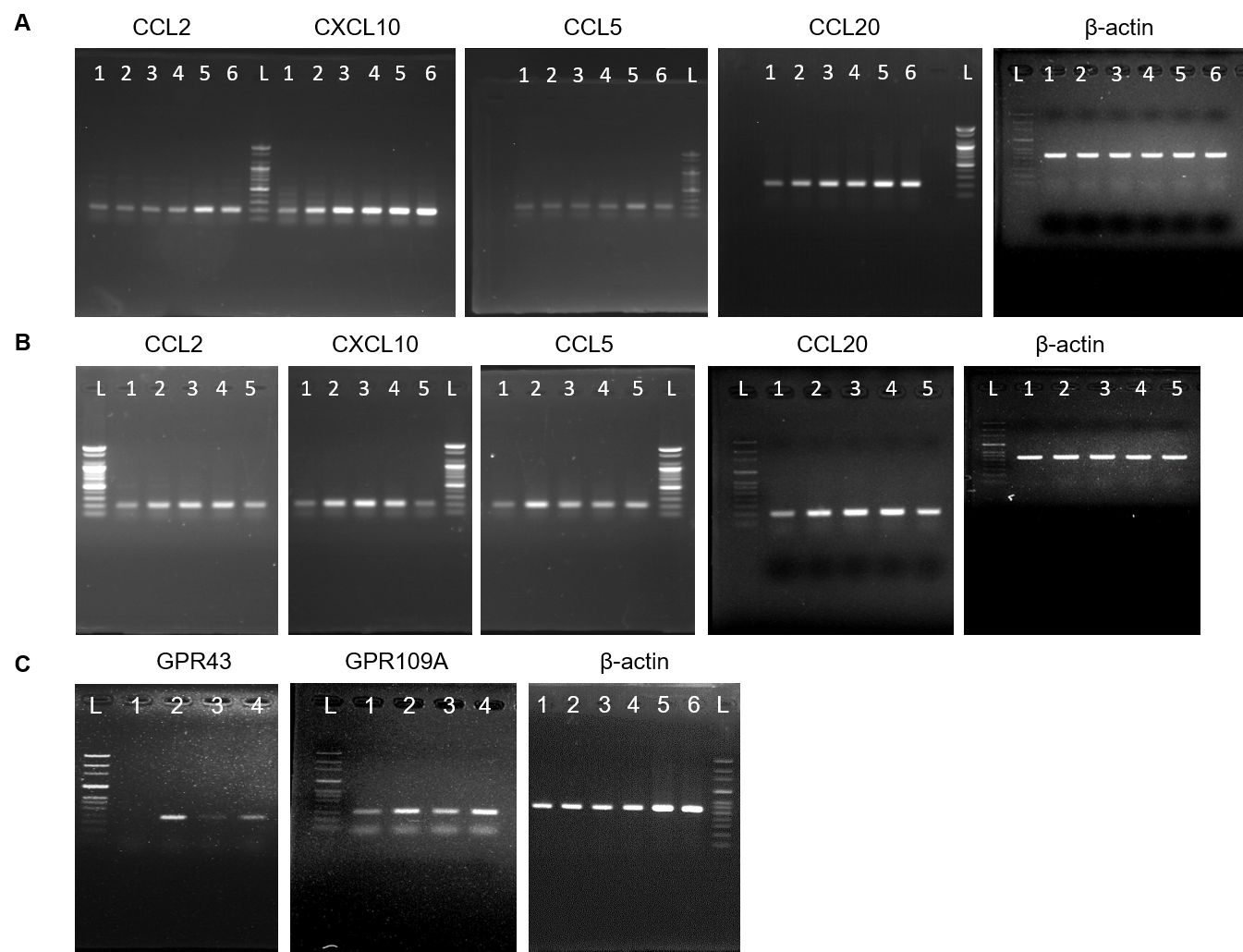
**

**Supplementary Figure 5 Entire gels for Figure 1A, 1B, and Figure 3A.**

Panel (A) corresponds to entire gels of Figure 1A of the manuscript. lane 1: Caco-2 cells without treatment; lane 2: Caco-2 cells treated with Shanchol^TM^ (10^6^ CFU/ml); lane 3: Caco-2 cells treated with Shanchol^TM^ (10^7^ CFU/ml); lane 4: Caco-2 cells treated with Shanchol^TM^ (10^8^ CFU/ml); lane 5: Caco-2 cells treated with Shanchol^TM^ (10^9^ CFU/ml); lane 6: Caco-2 cells treated with Pam2CSK4 (1 μg/ml); L: Ladder of size markers; Panel (B) corresponds to entire gels in the Figure 1B of the manuscript. lane 1: Caco-2 cells without treatment; lane 2: Caco-2 cells treated with Shanchol^TM^ (10^9^ CFU/ml) for 1 h; lane 3: Caco-2 cells treated with Shanchol^TM^ (10^9^ CFU/ml) for 3 h; lane 4: Caco-2 cells treated with Shanchol^TM^ (10^9^ CFU/ml) for 6 h; lane 5: Caco-2 cells treated with Shanchol^TM^ (10^9^ CFU/ml) for 12 h; L: Ladder of size markers; Panel (C) corresponds to gels of Figure 3A of the manuscript. lane 1: Caco-2 cells without treatment; lane 2: Caco-2 cells treated with Shanchol^TM^ (10^9^ CFU/ml); lane 3: Caco-2 cells treated with butyrate (10 mM); lane 4: Caco-2 cells co-treated with Shanchol^TM^ (10^9^ CFU/ml) and butyrate (10 mM); lane 5 and 6: loading control of THP-1 cells; L: Ladder of size markers.


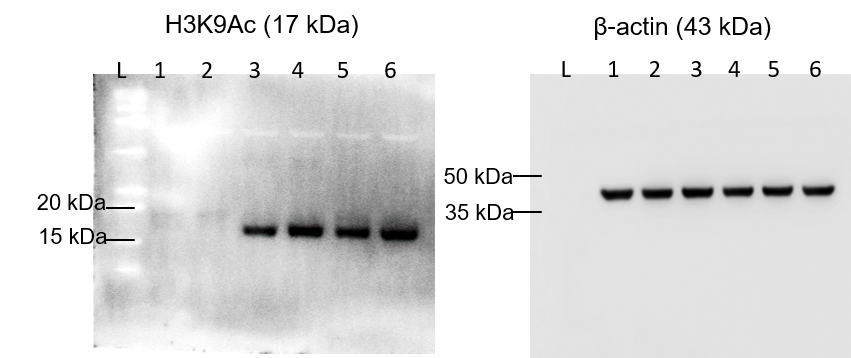


**Supplementary Figure 6 Complete blot images for the Figure 5D.**

Lane 1: Caco-2 cells without treatment; lane 2: Caco-2 cells treated with Shanchol^TM^ (10^9^ CFU/ml); lane 3: Caco-2 cells treated with butyrate (10 mM); lane 4: Caco-2 cells treated with trichostatin A (5 μM); lane 5: Caco-2 cells co-treated with Shanchol^TM^ (10^9^ CFU/ml) and butyrate (10 mM); lane 6: Caco-2 cells co-treated with Shanchol^TM^ (10^9^ CFU/ml) and trichostatin A (5 μM); L: Pre-stained ladder of size markers.


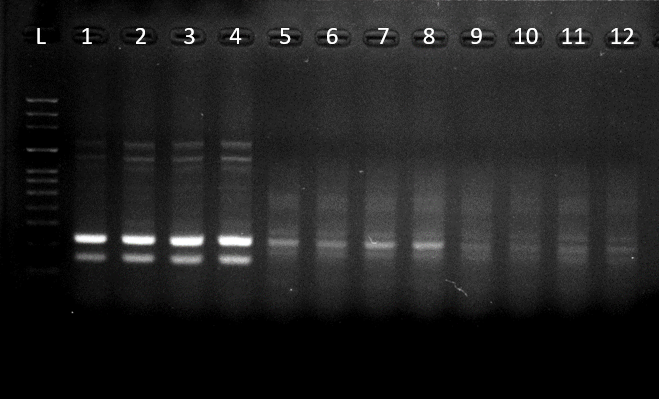


**Supplementary Figure 7** **Entire gel for Figure 5E.**

Input control (lanes 1 to 4), immunoprecipitated with acetylated histone H3 antibody (lanes 5 to 8) and immunoprecipitated with rabbit IgG isotype control antibody (lanes 9 to 12). lane 1: input control of Caco-2 cells without treatment; lane 2: input control of Caco-2 cells treated with Shanchol^TM^ (10^9^ CFU/ml); lane 3: input control of Caco-2 cells treated with butyrate (10 mM); lane 4: Input control of Caco-2 cells co-treated with Shanchol^TM^ (10^9^ CFU/ml) and butyrate (10 mM); lane 5: chromatin fragments of Caco-2 cells without treatment; lane 6 : chromatin fragments of Caco-2 cells treated with Shanchol^TM^ (10^9^ CFU/ml); lane 7: chromatin fragments of Caco-2 cells treated with butyrate (10 mM); lane 8: chromatin fragments of Caco-2 cells co-treated with Shanchol^TM^ (10^9^ CFU/ml) and butyrate (10 mM); lane 9: chromatin fragments of Caco-2 cells without treatment; lane 10: chromatin fragments of Caco-2 cells treated with Shanchol^TM^ (10^9^ CFU/ml); lane 11: chromatin fragments of Caco-2 cells treated with butyrate (10 mM); lane 12: chromatin fragments of Caco-2 cells co-treated with Shanchol^TM^ (10^9^ CFU/ml) and butyrate (10 mM); L: Ladder of size markers.


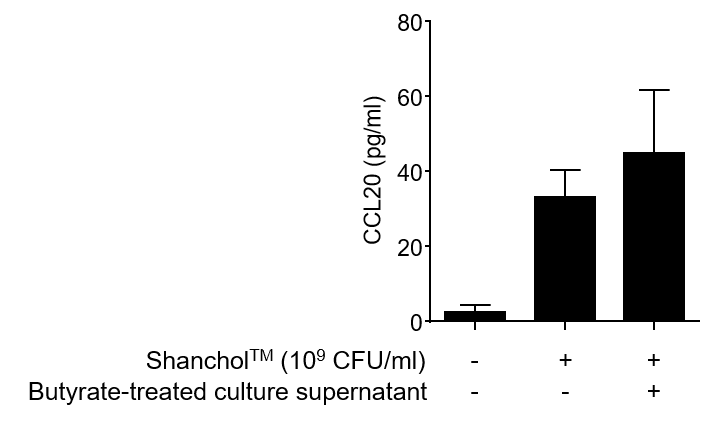


**Supplementary Figure 8 Culture supernatants of Caco-2 cells stimulated with butyrate does not enhance Shanchol^TM^-induced CCL20 secretion.**

Caco-2 cells were stimulated with Shanchol^TM^ (10^9^ CFU/ml) in the presence or absence of culture supernatants from Caco-2 cells treated with butyrate (10 mM) for 24 h. Then, the culture supernatants were collected, and the secreted CCL20 was measured by ELISA. All results are expressed as mean ± SD of triplicate samples.


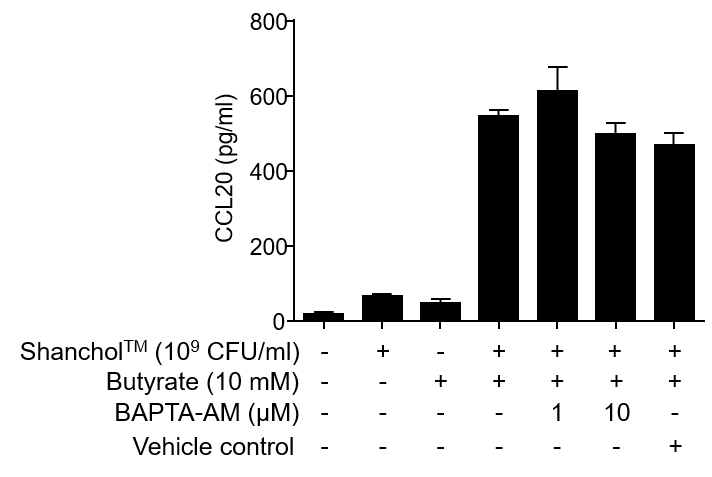


**Supplementary Figure 9 Ca^2+^ influx is not involved in the production of CCL20.**

Caco-2 cells were pre-treated with the indicated concentrations of BAPTA-AM or 0.1% DMSO (vehicle control) for 1 h. Then, the cells were co-stimulated with Shanchol^TM^ (10^9^ CFU/ml) and butyrate (10 mM) for an additional 24 h, the culture supernatants were collected, and the concentrations of secreted CCL20 were measured by ELISA. All results are expressed as mean ± SD of triplicate samples.
